# Screening of Healthy Feral Pigeons (*Columba livia domestica*) in the City of Zurich Reveals Continuous Circulation of Pigeon Paramyxovirus-1 and a Serious Threat of Transmission to Domestic Poultry

**DOI:** 10.3390/microorganisms10081656

**Published:** 2022-08-17

**Authors:** Désirée Annaheim, Barbara Renate Vogler, Brigitte Sigrist, Andrea Vögtlin, Daniela Hüssy, Christian Breitler, Sonja Hartnack, Christian Grund, Jacqueline King, Nina Wolfrum, Sarah Albini

**Affiliations:** 1 National Reference Center for Poultry and Rabbit Diseases, Institute for Food Safety and Hygiene, Vetsuisse Faculty, University of Zurich, 8057 Zurich, Switzerland; 2 Institute of Virology and Immunology (IVI), 3147 Mittelhäusern, Switzerland; 3 Department of Infectious Diseases and Pathobiology, Vetsuisse Faculty, University of Bern, 3012 Bern, Switzerland; 4 Gamekeeper, Grün Stadt Zürich, 8057 Zurich, Switzerland; 5 Section of Epidemiology, Vetsuisse Faculty, University of Zurich, 8057 Zurich, Switzerland; 6 Institute of Diagnostic Virology, Friedrich Loeffler-Institut, 17493 Greifswald, Germany

**Keywords:** Avian Orthoavulavirus-1, pigeon paramyxovirus-1, feral pigeon, *Columba livia domestica*, reverse transcriptase real-time PCR, Newcastle disease

## Abstract

Pigeon paramyxovirus-1 (PPMV-1) is predominantly isolated from pigeons or doves and forms a separate group of viral strains within Avian Orthoavulavirus-1, the causative agent of Newcastle disease in poultry. Since the introduction of PPMV-1 into Europe in 1981, these strains have rapidly spread all over Europe, and are nowadays considered to be enzootic in feral and hobby pigeons (*Columba livia domestica*). Infections with PPMV-1 can range from asymptomatic to fatal. To assess whether PPMV-1 continuously circulates in healthy feral pigeons, 396 tissue samples of pigeons from the city of Zurich were tested by reverse transcriptase real-time PCR over the period of one year. PPMV-1-RNA was detected in 41 feral pigeons (10.35%), determined as the dominant European genotype VI.2.1.1.2.2. In 38 of the 41 pigeons where organ samples tested positive, PPMV-1-RNA was also detected in either choana or cloaca swabs. There were no significant differences in positivity rates between seasons, age, and sex. The current study shows that feral pigeons without clinical signs of disease can harbour and most likely excrete PPMV-1. Spill-over into free-range holdings of chickens are therefore possible, as observed in a recent outbreak of Newcastle disease in laying hens due to PPMV-1 genotype VI.2.1.1.2.2. in the canton of Zurich in January 2022.

## 1. Introduction

Infections with a virulent strain of Avian Orthoavulavirus-1 (AOAV-1; formerly avian paramyxovirus APMV-1) [1,2] can cause Newcastle disease (ND) in poultry [3], one of the most important infectious diseases in chickens worldwide [4]. ND is defined by its virulence (“infection of poultry by an Avian Orthoavulavirus-1 that has an intracerebral pathogenicity index (ICPI) in day old chicks (*Gallus gallus*) of 0.7 or greater”), or a specific amino acid sequence at the fusion protein gene cleavage site (“multiple basic amino acids (positions 113–116) at the C-terminus of the F2 protein and phenylalanine at position 117 at the end of the F1 protein”) [3].

The known host spectrum of AOAV-1, however, is not restricted to chickens, but in fact includes more than 236 avian species, and all bird species are considered susceptible [5]. Within AOAV-1, pigeon strains consisting mostly of isolates derived from pigeons or doves, are named “pigeon paramyxovirus-1 (PPMV-1)” and cluster into genotype VI and XXI [1,6]. PPMV-1 likely arose around 1978/79 in the middle-east [7], and the first introduction of these strains into continental Europe was noted in Italy in 1981 in racing pigeons [8,9]. Between 1981–1984, PPMV-1 rapidly spread all over Europe, with the first cases in Switzerland noted in 1983 [9]. Today, PPMV-1 is considered to be enzootic in feral, racing, and fancy pigeons [10,11].

PPMV-1 is excreted via respiratory secretions, faeces, or urine of infected pigeons, and is transmitted horizontally between animals by inhalation or ingestion [12]. Pigeons show first clinical signs seven to fourteen days after infection with PPMV-1. These include neurological and intestinal signs with torticollis and greenish diarrhoea. An asymptomatic course of infection is also possible [12,13,14]. The mortality rate varies greatly between 10–70%, as does the morbidity between 30–80% [15]. Clinically healthy animals may shed virus and therefore represent a source for unperceived virus distribution [14].

PPMV-1 can thus also have an impact on domestic poultry if the virus is transmitted from infected pigeons or their droppings. Since its arrival in Europe, several European countries have been affected by ND outbreaks due to PPMV-1 in chicken. In the UK, feedstuff was contaminated by feral pigeons in the docks in Liverpool, resulting in 19 ND outbreaks due to PPMV-1 in chickens in 1984 [8]. Over 800,000 chickens were culled, and the financial loss amounted to more than 2.2 million pounds. Other outbreaks of PPMV-1-ND in commercial and backyard poultry were reported, for example, in Ireland [16], Germany [17], Sweden, France, Estonia [11], Macedonia [18], and in South Africa [19]. The virulence of PPMV-1 in chickens is generally intermediate, i.e., mesogenic [20,21], and may vary from subclinical to a drop in egg production in adult chickens [12,22]. Although the fusion protein gene cleavage site usually includes multiple basic amino acids, typical for a virulent NDV, some strains are lentogenic with an ICPI below 0.7 [20,23]. In addition, passage through chickens may result in an increased pathogenicity for chickens [20,24,25].

In Switzerland, Newcastle disease in poultry is a notifiable animal disease, and vaccination is prohibited. The national law requires poultry with positive serological or PCR findings to be culled [26]. In the past 10 years, only two outbreaks of genotype VII viruses occurred in laying hens [27]. Freedom of disease is monitored by testing free-range chickens and turkeys by serology. Contrary, PPMV-1 outbreaks with high morbidity and mortality among hobby or feral pigeons in Switzerland have occurred repeatedly in the past. The most noticed outbreaks in feral pigeons were 2011 and 2019 in the western part of Switzerland (cities of Geneva, La Chaux-de-Fonds), 2014 in the northern, eastern, and western part (cities: Schaffhausen, Chur, and Berne), and 2020 in the northern part (city of Basel) [28]. PPMV-1 infections in pigeons are also notifiable and, on average, two cases per year were reported to the Swiss authorities in the period from 1992–2021 [28]. In contrast to poultry, immunization for pigeons with inactivated vaccines is permitted and vaccination for competing racing pigeons is obligatory. Culling of individual pigeons or flocks found to be positive is not mandatory [26].

In PPMV-1 outbreaks with notable high mortality rates, veterinary authorities urge the poultry industry to be vigilant and the public to contact the gamekeeper and not to touch sick or dead pigeons. Although the zoonotic potential of AOAV-1, including PPMV-1, is considered to be low, direct contact with infected birds can lead to conjunctivitis or, rarely, to flu-like symptoms [29,30,31].

Few studies are available about virus carriage in asymptomatic feral pigeons [32,33,34,35]. However, feral pigeons shedding PPMV-1 while foraging in rural areas on the outskirts of cities poses a potential threat for the spread of the disease to free-range poultry holdings in Switzerland, as 84.5% of Swiss laying hens have access to a free-range area [36]. Keeping this in mind, the current study assessed the positivity rate, i.e., the proportion of positive samples among samples tested for PPMV-1, in the feral pigeon population of Zurich over the course of one year. The aims were further to investigate whether there are effects of virus carriage between seasons, and to collect data on the potential of PPMV-1 spreading to Swiss free-range poultry. Unexpectedly, while compiling the data of the current study, the first Newcastle disease outbreak in Switzerland due to PPMV-1 occurred in a flock of 500 laying hens, in a village roughly 13 km from the city centre of Zurich, in January 2022.

## 2. Materials and Methods

### 2.1. Samples of Feral Pigeons from the City of Zurich (Monitoring Samples)

The feral pigeon population management strategy in the city of Zurich is based on monitoring the number of feral pigeons and continuous culling by the gamekeeper. Samples were derived from these reduction efforts (birds were either caught with live cage traps and killed by manual cervical dislocation or shot by the gamekeeper). Each pigeon carcass was labelled with the date, location, and whether the bird showed any clinical signs.

A maximum of five pigeons from each location and collection date were assigned to the current study. Carcasses were kept at −20 °C until further processing and picked up monthly out of the storage freezer. Carcasses were collected in the period from 1 October 2020 to 30 September 2021, and seasons were assigned as follows: autumn (September until November), winter (December until February), spring (March until May), and summer (June until August). In total, 396 free-ranging feral pigeons (*Columba livia domestica*), originating from 21 different locations within the city of Zurich, were investigated. None of the birds were killed intentionally for this study.

After thawing, each pigeon was given an identification number. The location, the date of collection, age (juvenile or adult), sex (male or female), method of killing (cervical dislocation or shot), body condition (BC; good, moderate, or emaciated), and any gross pathological changes were noted, but not further investigated. Age was determined based on external attributes: juvenile pigeons have red legs and their cere (the fleshy, bulbous area around the nares right above the beak) is dark brown-red. In adults, the legs are dark red and the cere is powdery white. Presence of either ovary or testes was checked for females or males, which was possible for all but 13 pigeons, where tissue damage inflicted by shooting did not allow for sex determination. The detection of lesions due to shooting or cervical dislocation affirmed the cause of death. Subsequently, one swab sample each from choana and cloaca, and tissue samples (kidney and brain) were collected. The samples were frozen at −80 °C until further use. In order to follow a resource-efficient approach, initially only one “primary sample” per pigeon was processed. This primary sample was generally kidney, but in cases where kidney was not available due to severe damage of the carcass from shooting (*n* = 5), alternatively, brain was the primary sample.

### 2.2. Samples of Laying Hens from the Newcastle Disease Outbreak Due to PPMV-1, January 2022 (Outbreak Samples)

In a flock of 500 laying hens (*Gallus gallus domesticus*), laying performance (daily egg production) was reduced by >60% within one day, and with an abundance of shell-less eggs. The animals showed no respiratory, intestinal, or neurological signs, and no mortality. The laying hens had access to a free-range area but were kept indoors after the onset of the disease. Twenty serum samples were tested for Newcastle disease, Avian Influenza and Egg Drop Syndrome 1976 by ELISA (all from Biochek, Reeuwijk, The Netherlands) and 60 choana-cloaca swab samples were examined by AOAV-1 real-time RT-PCR (rRT-PCR; see below) and Avian Influenza rRT-PCR, as described elsewhere [37]. Additionally, organ samples from hens (V-0043; hens No 1, 3, 7, 9) and two culled feral pigeons (V-0051) from the farm premises were also examined.

### 2.3. RNA Extraction from Tissue and Swab Samples

RNA was extracted from kidney, brain, and swabs using a commercial kit (RNA purification from tissue, NucleoSpin^®^ RNA; Macherey-Nagel, Dueren, Germany) according to the manufacturer’s instructions (for swabs, the homogenization was omitted). If the primary organ sample was positive for AOAV-1 by rRT-PCR, RNA was also extracted from brain and from choanal and cloacal swabs. To control the efficiency of RNA extraction and to detect potential inhibitory effects, an internal control consisting of in vitro transcribed eGFP RNA was added to the samples prior to extraction, as described elsewhere [38].

### 2.4. Real-Time Reverse Transcriptase PCR (rRT-PCR) for the Detection of AOAV-1

All primers and probes used in this study are listed in Table 1. For detection of the AOAV-1 matrix and fusion protein genes (M- and F-gene) the rRT-PCR assay described by Wise et al., 2004 [39], was used in combination with an internal control (eGFP RNA) according to Hoffmann et al., 2005 [40]. For the rRT-PCR, a one-step protocol was applied using the one-step QuantiTect Probe RT-PCR kit (Qiagen, Hilden, Germany). For 25 µL reaction mixture 12.5 µL “QuantiTect Probe RT-PCR Mastermix [end concentration: 1×]”, 0.75 µL [300 nM] of each forward and reverse primer, 0.625 µL [250 nM] of the probe, 0.25 µL QuantiTect RT Mix, DEPC treated water (5.125 µL M-gene specific assay, 2.875 µL F-gene specific assay), and 5 µL sample RNA were used. 

For detection of the internal control (eGFP gene), 1 µL [400 nM] of the forward and reverse primer and 0.25 µL [100 nM] of the probe were added to the F-gene specific rRT-PCR mix. 

rRT-PCR was performed on the “Applied Biosystem 7500-Fast” with the following conditions: 48 °C for 30 min, 95 °C for 10 min followed by 45 cycles of 15 s at 95 °C, 1 min at 53 °C, and 1 min at 72 °C.

All samples were tested in duplicates. RNA of a characterized field strain of Newcastle disease (G1227 (CH-1/95); [41]) was used as positive control, and molecular grade water as a negative control.

If no target RNA was amplified, the pigeon samples were classified as negative. If the mean cycle threshold (C_T_ value) was <36 in either the NDM or NDF rRT-PCR, the samples were classified positive for AOAV-1. Samples with C_T_ values > 36 were re-tested with both the NDM and NDF rRT-PCR assays. Samples were positive in the retesting, with either PCR considered positive. If the primary sample of a pigeon was positive, rRT-PCRs were subsequently performed using RNA extracted from brain tissue, choanal, and cloacal swabs.

### 2.5. Sequencing of the F- and HN-Gene

For sequencing of the F- and HN-gene, the samples were initially amplified with the respective primer pairs (Table 1) to obtain four PCR fragments per sample. After subsequent pooling of the four PCR fragments, a purification step with AMPure XP Magnetic Beads (Beckman-Coulter, Brea, CA, USA) in a bead to sample volume ratio of 0.65X was conducted. Sequencing utilizing the Mk1C MinION platform (Oxford Nanopore Technologies, ONT, Oxford, UK) in combination with the Rapid Barcoding Kit (SQK-RBK004, ONT) for sample multiplexing and a R9.4.1 flow cell was performed according to the manufacturer’s instructions. Live basecalling of the raw data was implemented with Guppy (v.4.3.4, ONT), followed by a demultiplexing, quality check, and trimming step to remove all primer and low-quality sequences. Generation of the consensus sequences was achieved in a map-to-reference approach with MiniMap2 [42]. The software Geneious Prime (Biomatters, Auckland, New Zealand) was used for polishing of the final genome sequences. Data included in this study were deposited in the NCBI GenBank database (chickens: ON986198-ON986201; pigeons: ON986202-ON986206).

For further analysis, sequences were aligned using ClustalW and analyzed in MEGA 10.0.5 [43] with either a data set representing a pilot collection of all NDV genotypes or with a collection of genotype VI viruses as previously presented [1]. The phylogenetic analysis was performed by maximum likelihood analysis (1000 bootstrap replicates) and Tamura 3-parameter as the best-fitted Model.

### 2.6. Statistics

Statistical analyses were carried out using R version 4.1.2 software [44]. For the descriptive statistical analysis, 9% binomial confidence intervals, according to Jeffreys, were determined with the R package DescTools [45].

The 13 pigeons, where the sex could not be determined, were assigned to the sex which was most represented for calculation (i.e., female). To assess if the four factors (season, being adult or juvenile, sex, and mode of killing) were significantly associated with positive or negative test results, a generalised mixed effects model with the four factors as fixed effects and geographical location (ZIP code) as random effects were performed with the package lme4 [46].

## 3. Results

### 3.1. PPMV-1 in Feral Pigeons from the City of Zurich

AOAV-1-RNA was detected in 41 primary samples from 396 feral pigeons (10.35%; 95% confidence interval CI, 7.64–13.64%). AOAV-1-RNA was not detected in any of the pigeons where brain was the primary sample (*n* = 5). Of the 41 pigeons with M-gene positive kidney samples, 12 kidney samples were also positive by the F-gene rRT-PCR (3.03%, CI, 1.67–5.07%). Furthermore, 31 brain samples (75.61%, CI, 61.03–86.71%) and swabs from 38 pigeons (92.68%, CI, 81.74–97.89%) were positive for the M-gene and/or for the F-gene (Table A1). From 22 pigeons (53.66%, CI, 38.58–68.23%), AOAV-1-RNA was detected in both choana and cloacal swabs. In nine (21.95%, CI, 11.45–36.24%) and seven (17.07%, CI, 7.97–30.62%) pigeons, respectively, it was detected only in the cloacal swabs or in the choanal swabs (Table A1).

**Figure 1:**
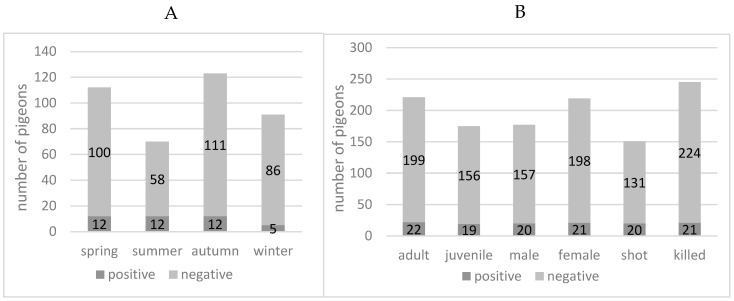
Positivity rate (Avian Orthoavulavirus-1 M-gene rRT-PCR) of feral pigeons (*Columba livia domestica*) from the city of Zurich. (**A**) In different seasons. (**B**) According to age, sex, and mode of death.

The cross-classified proportions of positive test results by season, age category, sex, and mode of killing are presented in Figure 1. Numbers of PPMV-1 positives pigeons per total examined are: (i) according to season, spring 12/112 (10.71%, CI, 5.99–17.43%), summer 12/70 (17.14%, CI, 9.73–27.14%), autumn 12/123 (9.76%, CI, 5.44–15.93%), and winter 5/91 (5.49%, CI, 2.13–11.62%); (ii) to age, adult 22/221 (9.95%, CI, 6.53–14.42%), or juvenile 19/175 (10.86%, CI, 6.89–16.10%); (iii) to sex, male 20/177 (11.30%, CI, 7.27–16.58%), or female 21/219 (9.59%, CI, 6.22–14.02%); and (iv) mode of death, shot 20/151 (13.25%, CI, 8.55–19.33%), or killed 21/245 (8.57%, CI, 5.55–12.57%). Based on the 95% confidence intervals, there is no evidence of a significant association with any of the four factors. Similarly, based on the generalised mixed effects model, there was no evidence for a significant association (data not shown).

**Figure 2:**
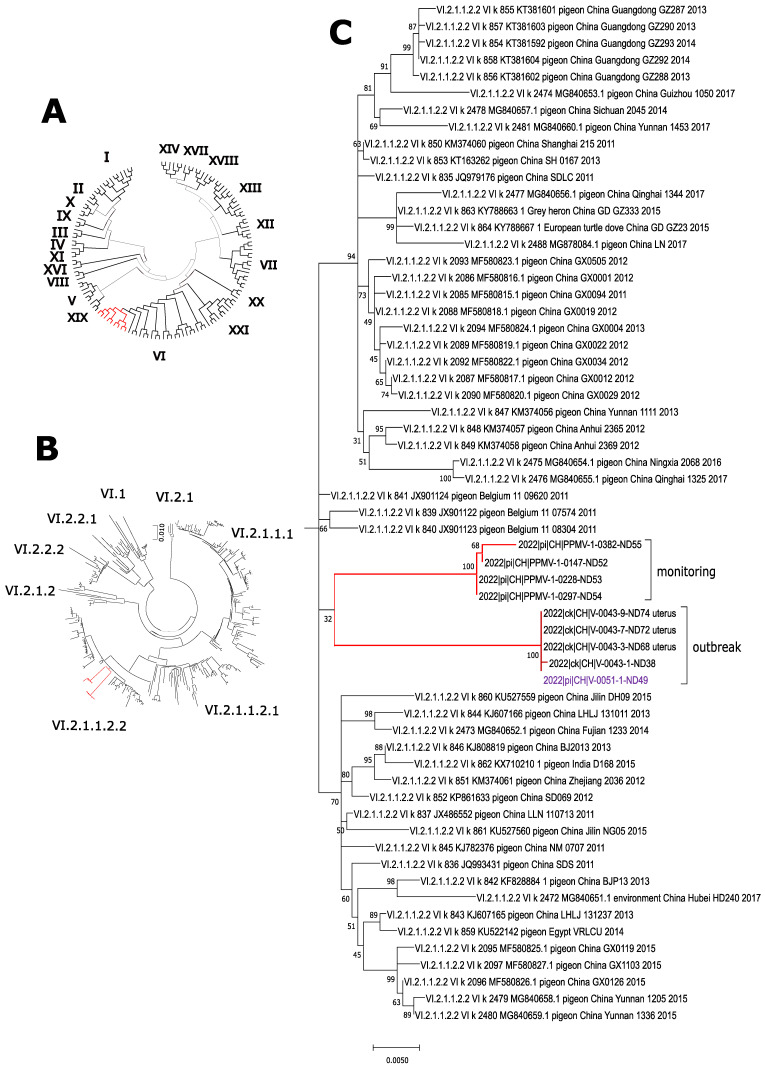
Phylogenetic analysis of detected PPMV-1. Fusion gene sequences obtained from the feral pigeon monitoring programme and from an outbreak in laying hens (indicated by red bars) clustered within genotype VI when analyzed using a pilot collection of all genotypes (**A**). Further analyses with a collection of genotype VI viruses grouped all viruses to subgenotype VI.2.1.1.2.2 (**B**), forming a separate branch within this subgenotype (**C**), closely related to European viruses.

Phylogenetic analysis of the F-gene of four kidney samples of selected pigeons (one from each season; P0147, P0228, P0297, P0382) revealed that the viruses belong to genotype VI of class 2 AOAV-1, confirming the assumed PPMV-1 origin (Figure 2). Within this genotype, all viruses grouped within subgenotype VI.2.1.1.2.2. (Figure 2B, Table A1), forming a specific branch most closely related to viruses from Europe (Figure 2C). It is interesting to note that viruses from winter 2020/21 (P-0147), spring (P-0228), and summer 2021 (P-0297) were almost identical, whereas the sample from autumn 2021 (P-0382) was slightly divergent, with seven nucleotides exchanges, respectively (Table 2) (Data of the sequencing of the HN-gene are not shown).

In total, 382 of the feral pigeons examined were clinically healthy, as observed by the gamekeeper, and no pathologies were noted during macroscopic examination in the necropsy. The gamekeeper reported that three pigeons were found apathic (P-134, P-218, P-382). Two (P-134 and P-382) of these showed a moderate body condition (BC). In P-382, abundant miliary white spots were observed in the kidney during necropsy. Two pigeons (P-21 and P-50) had crusty proliferations on beak and legs compatible with pigeon-pox infection. One of these (P-21) had a moderate BC. A further seven pigeons had moderate BC without other findings. In one pigeon, (P-281) greenish diarrhoea was observed. However, from the above-mentioned pigeons with clinical or necropsy findings, only two (P-218 and P-382) were positive for PPMV-1 by rRT-PCR (Table A1).

### 3.2. Newcastle Disease Outbreak in Laying Hens Due to PPMV-1, January 2022

In January 2022, serum samples from a flock of laying hens that had suffered a severe drop in egg production of >60% tested positive for NDV antibodies by ELISA, with 15 out of 20 samples being highly positive (data not shown). Further serological testing by ELISA gave no indication of an infection with Avian Influenza virus or Egg Drop Syndrome 1976 virus (Duck Atadenovirus A). Subsequent testing of combined choanal and cloacal swabs by AOAV-1 rRT-PCR revealed that 50 out of 60 samples were positive for AOAV-1-RNA. Two of the feral pigeons, which were found in large numbers on the farm, were examined, and both kidney samples tested positive for PPMV-1. Sequence analysis of samples from laying hens (V-0043) and one feral pigeon (V-0051) from the outbreak hold revealed identical sequences; both are genotype VI.2.1.1.2.2 viruses like in the samples from the monitoring programme (Figure 2). However, these viruses are forming a distinct branch within the subgenotype, with a diversity between 0.0366 to 0.412 to viruses from the monitoring programme (Table 2).

## 4. Discussion

The current study investigated a possible circulation of PPMV-1 in clinically healthy feral pigeons in Zurich. In a total of 396 samples taken between October 2020 to September 2021, AOAV-1-RNA was detected in 41 feral pigeons (Figure 1). First, kidney samples were examined, because this organ was known to be reliably positive in case of an infection [47], and a sufficient amount of organ material could be harvested at necropsy for downstream analyses. From 41 pigeons testing positive in the primary organ sample, collected swab samples were subsequently tested, where 38 out of 41 pigeons were also positive for PPMV-1 in either choana, cloaca, or choana and cloaca (Table A1). Thus, there is an indication that a large proportion of the PPMV-1 positive pigeons shed the virus. However, based on the detection of RNA, no statement can be made about the shedding of viable viruses. 

Compared to other studies, the positivity rate of 10.35% (95%, CI, 7.64–13.64%) in 396 Zurich city pigeons is rather high (Figure 1). A study from the United States found 15 out of 1416 (1.03%) feral pigeons to be positive by M-gene rRT-PCR of brain [32]. Two further studies from Norway (200 cloacal swabs tested by rRT-PCR) [34] and Germany (241 kidney samples tested by rRT-PCR) [35] did not find any PCR-positive samples, but the German survey showed a prevalence of 40–51% based on serological data. Finally, a Japanese study collected 1021 pigeon droppings over the course of two years, but only one sample was positive for PPMV-1 (0.01%) by virus isolation [33]. Overall, the studies are difficult to compare due to differences in sample material and/or detection method.

Four samples of the current study (one out of each season) were shown to belong to genotype VI.2.1.1.2.2. (Figure 2B, Table A1). This is in accordance with the currently predominantly circulating genotype within the European pigeon population. Viruses/viral RNA detected from 1983 onwards grouped mostly within genotype VI.2.1.1.2. according to the new nomenclature [1,2], or genotype VIb according to the previous nomenclature [1,6,41]. However, genotype XXI.1.1 (formerly genoytpe VI g [48]) seems to co-circulate to a lesser extent in pigeons in Germany [49].

According to the gamekeeper, the Zurich feral pigeon population is clinically healthy. Most of the examined pigeons did not show any clinical signs, such as weakness or neurological signs, and no larger mortality events were observed during the study period. Just two of the PPMV-1 positive pigeons (P-218, P-382) were found to be apathic at collection by the gamekeeper, and just one of these had gross pathological changes (P-382) (Table A1). These (miliary spots in the liver) were not indicative of a PPMV-1 infection, but rather pointed to a concomitant bacterial infection. All other 39 PPMV-1 positive pigeons showed no clinical signs, had a good body condition, and had no gross pathological changes (Table A1). Nevertheless, conclusions on pathogenic potential for the feral pigeons are not possible. Due to the design of the study, it remains unclear whether some pigeons underwent a subclinical infection as known from infection experiments with racing pigeons or whether PPMV-1 positive pigeons were at the stage of an incubation period and would have develop clinical disease later [12]. In addition, it cannot be excluded that sick pigeons are not represented in the sample material, because they may be hiding or may have fallen prey to predators, such as dogs, cats, foxes, or birds of prey. Even though sequencing of the fusion protein cleavage site revealed an amino acid sequence of “RRQKRF” indicative of a virulent virus, this may not correspond to a meso-/velogenic pathotype in pigeons [20,23]. Regardless of the individual clinical course, the lack of typical macroscopic lesions like necrotic foci in the pancreas in conjunction with the field observations, our study clearly showed enzootic, clinical unnoticed circulation of PPMV-1 in feral pigeons.

There was no significant difference in positivity rates of adult or juvenile male or female pigeons (Figure 1B). The method of capture or killing was recorded to check whether PPMV-1-positive animals might be easier to shoot or easier to catch, but the mode of death did not correlate with the positivity rate (Figure 1B). Two-thirds of the positive pigeons were found in summer and spring, and one-third in winter and autumn, however the differences were not significant (Figure 1A). The opposite pattern was observed by the above-mentioned study in the USA [32], where two-thirds of the 10/15 positive animals were found in winter and one-third (5/15) in summer. The authors suggested that the warm weather had a negative influence on the stability of the virus, but the current study could not support this hypothesis.

Although the location of each collected feral pigeon was recorded by the gamekeeper, an analysis comparing the 21 different location was not done (Table A1). The reason for this is that it was not possible to determine whether each of these sites represented a unit without influxes from other pigeon groups, as no data were available on the usual residence area of the individual pigeons. Generally, pigeons prefer to stay very close to their nest, but studies examining the home range of feral pigeons obtained various results between 0.3 and 25 km [50,51,52]. Furthermore, since the current study was carried out during the ongoing human COVID-19 pandemic—in which lockdowns or working from home meant reduced human presence in public, resulting in less food scraps in the city—the gamekeeper observed that the feral pigeons extended their usual range to rural areas in search of food.

Management of feral pigeons differ from city to city in Switzerland. In Basel, the feral pigeon population was reduced from approximately 20,000 to 10,000 birds within 50 months by banning public feeding, educating the residents, and establishing pigeon lofts. [53]. Further lofts were established, e.g., in the cities of Berne, Lucerne, and Chur. Lofts provide food and sheltered nests, where eggs can also be replaced by dummies [53]. Furthermore, Berne also started a vaccination programme following a PPMV-1 outbreak with high mortality in 2014/2015. In Zurich, population control is done by annual culling of approximately 1500 individuals by the gamekeeper. A previous study [54] investigating the prevalence of *Chlamydia (C.) psittaci* and *C. avium* in pigeons of different Swiss cities found the occurrence in feral pigeons from the Greater Zurich area to be almost twice as high as in the loft managed Lucerne area and the city of Berne. It has been suggested that culling leads to a constant restructuring of the population, as culled individuals are compensated for by new mates and juveniles. This results in greater mingling or increased contact between pigeons [52]. Contrary, pigeon populations managed in loft systems seem to result in a smaller, healthier, and more stable population [53,54]. It remains speculative, but the high positivity rate of PPMV-1 in the Zurich feral pigeons compared to the afore mentioned feral pigeon studies, could possibly also be linked to the culling control strategy. However, the Zurich feral pigeons were forced to expand their action radius due to the human COVID-19-pandemic-related measures, which resulted in fewer feeding opportunities. This probably also resulted in more mixing of pigeons, which may indirectly account for the high PPMV-1 positivity rate observed in this study. 

The expansion of the foraging grounds to rural areas was thought to increase the risk of PPMV-1 transmission to free-ranging poultry. Surprisingly, while compiling the data of the pigeon study, such a ND outbreak indeed happened in January 2022. A drop in egg production of >60% within one day was perceived in a flock of 500 free-range laying hens. Chickens were found to be positive for AOAV-1 by serology and rRT-PCR. Sequencing revealed genotype VI.2.1.1.2.2, the currently circulating PPMV-1 genotype in Europe (Figure 2B). The source of infection was a large number (>200 individuals) of apparently healthy feral pigeons present on the farm premises and on the surrounding land and feeding on easily accessible feed from suckler cows next to the free-range area of the laying hens. Analysis of the outbreak viruses of the hens and a pigeon showed them to be identical (Table 2, Figure 2C). The ND outbreak was thus proven to be an incursion of a wild bird virus. The affected chickens were culled, and spill-over from the index farm into other farms did not occur. All samples tested from the protection zone and surveillance zone were negative.

## 5. Conclusions

Continuous circulation of PPMV-1 could be demonstrated in asymptomatic feral pigeons. A possible solution to minimise the risk of transmission to poultry is to manage the feral pigeon population to create a stable and healthy pigeon population, thereby reducing virus shedding.

## Figures and Tables

**Table 1:** Primers and probes for the AOAV-1 real-time RT-PCR assay used in this study.

Method	Target	Primers and Probes	Sequence (5′-3′)	Amplicon Size (bp)	Ref.
Avian Orthoavulavirus-1 (AOAV-1) real-time RT-PCR	M-protein gene	M+4100	AGT GAT GTG CTC GGA CCT TC	121	[39]
		M-4220	CCT GAG GAG AGG CAT TTG CTA		
		M+4169	FAM-TTC TCT AGC AGT GGG ACA GCC TGC-TAMRA		
	F-protein gene	F+4839	TCC GGA GGA TAC AAG GGT CT	101	
		F-4939	AGC TGT TGC AAC CCC AAG		
		F+4894	FAM-AAG CGT TTC CTC CTT CCT CCA-TAMRA		
	eGFP	eGFP1-F	GAC CAC TAC CAG CAG AAC AC	132	[40]
		eGFP2-R	GAA CTC CAG CAG GAC CAT G		
		eGFP1-Hex	HEX-AGC ACC CAG TCC GCC CTG AGC A-BHQ1		
Amplification of fusion (F)- and hemagglutinin-neuraminidase (HN)-gene fragments for sequencing	F-protein gene	PPMV-1_fw4283	CAA GCT GGC ACC CAA CGT	1644	
		PPMV-1_rev5927	TGC CTG TCA CGA TGA CTT GAG ART C		
	F-protein gene	PPMV-1_fw5369	TAT AYT GTA TGA CTC ACA GAC TC	944	
		PPMV-1_rev6340	CCG TTC TAC CCG TGB ATT GC		
	F-protein gene	PPMV-1_fw5841	GGA UAA CYC URA GGC UCA G	1797	
		PPMV1_rev7638	GGTACAAGAARTGAGAYGTCCCTA		
	HN gene	PPMV1_fw7219	CACATYAATGGTGCACGGAAG	1306	
		PPMV-1_rev8524	CTG CTG ATA AYG AGA TGR TCR		

bp, base pairs.

**Table 2:** Estimates of Evolutionary Divergence between Swiss PPMV-1 viruses ^1^.

			1	2	3	4	5	6	7	8	9	10	11
	1	JX901124|pi|BE|2011|09620											
	2	JX901122|pi|BE|2011|07574	0.0054										
	3	JX901123|pi|BE|2011|08304	0.0054	0.0061									
monitoring	4	2022|pi|CH|PPMV-1-**0228**-ND53	0.0184	0.0190	0.0202								
	5	2022|pi|CH|PPMV-1-**0147**-ND52	0.0190	0.0196	0.0209	0.0006							
	6	2022|pi|CH|PPMV-1-**0297**-ND54	0.0184	0.0190	0.0202	0.0000	0.0006						
	7	2022|pi|CH|PPMV-1-**0382**-ND55	0.0227	0.0234	0.0246	0.0042	0.0036	0.0042					
outbreak	8	2022|ck|CH|**V-0043**-3-ND68_uterus	0.0252	0.0271	0.0284	0.0366	0.0373	0.0366	0.0412				
	9	2022|ck|CH|**V-0043**-7-ND72_uterus	0.0252	0.0271	0.0284	0.0366	0.0373	0.0366	0.0412	0.0000			
	10	2022|ck|CH|**V-0043**-9-ND74 uterus	0.0252	0.0271	0.0284	0.0366	0.0373	0.0366	0.0412	0.0000	0.0000		
	11	2022|ck|CH|**V-0043**-1-ND38	0.0258	0.0277	0.0290	0.0373	0.0379	0.0373	0.0418	0.0006	0.0006	0.0006	
	12	2022|pi|CH|**V-0051**-1-ND49	0.0252	0.0271	0.0284	0.0366	0.0373	0.0366	0.0412	0.0000	0.0000	0.0000	0.0006

pi, pigeon; ck, chicken; BE, Belgium; CH, Switzerland; ^1^: Number of base substitutions per site. Analyses were conducted using the Tamura 3-parameter model.

## Data Availability

All sequencing data included in this study has been deposited in the NCBI GenBank Database (ON986198-ON986206). Data is contained within the article.

## Appendix A

**Table A1:** Examination of kidney, brain and swab samples from feral pigeons (*Columba livia domestica*) from the city of Zurich by M- and F-gene specific Avian Orthoavulasvirus-1 rRT-PCR. a = adult, j = juvenile; m = male, f = female; k = kidney, b = brain, ch = choana, cl = cloaca, neg = negative, pos = positive, n.a. = not analyzed, BC = body condition.

	Pigeon Carcass Data					Matrix (M)-Gen rRT-PCR				Fusion (F)-Gen r RT-PCR			
Season	Sample Nr.	Collection Data (Date/Location)		Age/ Sex	Observed Pathologies	Organs		Swabs		Organs		Swabs	
		Date (dd.mm.yy)	ZIP Code			k	b	ch	cl	k	b	ch	cl
winter	P 0115	22.01.21	8005	a/m	none	pos	pos	neg	neg	neg	pos	neg	neg
	P 0116	25.01.21	8051	j/f	none	pos	pos	neg	pos	neg	pos	pos	pos
	**P 0147 ^1^**	24.02.21	8005	a/m	none	pos	pos	pos	pos	pos	pos	neg	pos
	P 0148	24.02.21	8005	a/f	none	pos	pos	pos	pos	pos	pos	pos	pos
	P 0149	24.02.21	8005	a/f	none	pos	pos	pos	pos	pos	pos	pos	neg
spring	P 0172	30.03.21	8047	j/m	none	pos	pos	pos	pos	neg	neg	pos	pos
	P 0212	18.04.21	8005	a/f	none	pos	neg	pos	neg	neg	neg	pos	neg
	P 0217	14.04.21	8050	a/f	none	pos	pos	neg	neg	neg	pos	neg	neg
	P 0218	26.04.21	8047	j/m	apathic	pos	pos	pos	n.a.	pos	pos	pos	n.a.
	P 0227	01.04.21	8052	a/f	none	pos	pos	pos	pos	pos	pos	pos	pos
	**P 0228 ^1^**	01.04.21	8052	a/m	none	pos	pos	neg	pos	pos	pos	neg	pos
	P 0230	01.04.21	8052	j/m	none	pos	pos	pos	pos	pos	neg	neg	neg
	P 0234	07.04.21	8051	a/m	none	pos	pos	pos	neg	neg	neg	pos	pos
	P 0236	07.04.21	8051	a/m	none	pos	neg	pos	neg	neg	neg	neg	neg
	P 0259	28.05.21	8047	a/m	none	pos	neg	neg	pos	neg	neg	neg	neg
	P 0261	28.05.21	8047	j/f	none	pos	pos	pos	pos	neg	neg	neg	neg
	P 0263	20.05.21	8004	a/m	none	pos	pos	pos	pos	pos	neg	pos	pos
summer	P 0286	01.06.21	8047	a/f	none	pos	pos	pos	neg	neg	neg	neg	neg
	P 0289	01.06.21	8047	a/m	none	pos	pos	neg	pos	neg	neg	neg	neg
	P 0292	16.06.21	8038	a/m	none	pos	pos	neg	pos	neg	neg	neg	neg
	P 0293	16.06.21	8038	j/f	none	pos	pos	neg	pos	neg	neg	neg	neg
	P 0296	29.06.21	8005	j/f	none	pos	pos	pos	neg	pos	neg	neg	neg
	**P 0297 ^1^**	07.06.21	8004	j/f	none	pos	pos	pos	pos	pos	neg	neg	neg
	P 0305	30.06.21	8038	a/m	none	pos	pos	pos	pos	pos	neg	neg	neg
	P 0306	30.06.21	8038	a/f	none	pos	neg	pos	pos	neg	neg	neg	neg
	P 0311	07.06.21	8044	j/m	none	pos	pos	pos	pos	pos	neg	neg	neg
	P 0315	23.07.21	8001	j/f	none	pos	pos	pos	pos	neg	neg	neg	neg
	P 0316	23.07.21	8001	j/f	none	pos	neg	pos	pos	neg	neg	neg	neg
	P 0345	23.08.21	8001	j/m	none	pos	pos	pos	pos	neg	neg	neg	neg
autumn	P 0352	15.09.21	8001	a/m	none	pos	neg	pos	pos	neg	neg	neg	neg
	P 0355	27.09.21	8001	j/f	none	pos	pos	pos	pos	neg	neg	neg	neg
	P 0359	27.09.21	8001	j/m	none	pos	pos	neg	pos	neg	neg	neg	neg
	P 0360	20.09.21	8001	j/f	none	pos	pos	pos	pos	neg	neg	neg	neg
	P 0366	21.09.21	8048	a/?	none	pos	pos	neg	neg	neg	neg	neg	neg
	P 0368	21.09.21	8048	j/f	none	pos	pos	neg	neg	neg	neg	neg	neg
	P 0369	21.09.21	8048	j/f	none	pos	pos	pos	pos	neg	neg	neg	neg
	P 0371	15.09.21	8002	a/m	none	pos	neg	neg	pos	neg	neg	neg	neg
	P 0374	15.09.21	8002	a/f	none	pos	neg	pos	pos	neg	neg	neg	neg
	P 0377	13.09.21	8001	a/f	none	pos	neg	pos	neg	neg	neg	neg	neg
	P 0379	13.09.21	8001	j/m	none	pos	pos	neg	pos	neg	neg	neg	neg
	**P 0382 ^1^**	02.09.21	8006	j/m	apathic, moderate BC	pos	pos	pos	pos	neg	pos	neg	pos

^1^ for sample numbers in bold the genotype was determined to be VI.2.1.1.2.2 by sequencing of the fusion protein gene.
